# Comparative efficacy and safety of SGLT2is and ns-MRAs in patients with diabetic kidney disease: a systematic review and network meta-analysis

**DOI:** 10.3389/fendo.2024.1429261

**Published:** 2024-07-04

**Authors:** Si-Qi Yang, Xi Zhao, Jing Zhang, Huan Liu, Yu-Han Wang, Yao-Guang Wang

**Affiliations:** ^1^ Department of Nephrology, First Teaching Hospital of Tianjin University of Traditional Chinese Medicine, Tianjin, China; ^2^ Department of Nephrology, National Clinical Research Center for Chinese Medicine Acupuncture and Moxibustion, Tianjin, China; ^3^ Graduate School, Tianjin University of Traditional Chinese Medicine, Tianjin, China

**Keywords:** diabetic kidney disease, sodium-glucose cotransporter-2 inhibitor, non-steroid mineralocorticoid receptor antagonists, network meta-analysis, cardiorenal

## Abstract

**Objectives:**

To evaluate the efficacy and safety of non-steroid mineralocorticoid receptor antagonists (ns-MRAs) and sodium-glucose cotransporter 2 inhibitors (SGLT2is) in patients with diabetic kidney disease (DKD).

**Methods:**

Systematic literature searches were performed using PubMed, Embase and Web of Science encompassing inception until January 20, 2024. Randomized control trials (RCTs) comparing ns-MRAs and SGLT2is in DKD were selected. The efficacy outcomes of interest included kidney-specific composite outcome, cardiovascular (CV)-specific composite outcome, end-stage kidney disease (ESKD), and overall mortality. We also investigated safety outcomes, including acute kidney injury (AKI) and hyperkalemia.

**Results:**

A total of 10 randomized clinical trials with 35,786 patients applying various treatments were included. SGLT2is (SUCRA 99.84%) have potential superiority in kidney protection. SGLT2is (RR 1.41, 95%CI 1.26 to 1.57) and ns-MRAs (RR 1.17, 95% CI 1.08 to 1.27) were associated with significantly lower kidney-specific composite outcome than the placebo. Regarding the reduction in CV-specific composite outcome and ESKD, SGLT2is (SUCRA 91.61%; 91.38%) have potential superiority in playing cardiorenal protection. Concerning the CV-specific composite outcome (RR 1.27, 95%CI 1.09 to 1.43) and ESKD (RR 1.43, 95%CI 1.20 to 1.72), SGLT2is significantly reduced the risks compared to placebo. Regarding the reduction in overall mortality, SGLT2is (SUCRA 83.03%) have potential superiority in postponing mortality. Concerning the overall mortality, SGLT2is have comparable effects (RR 1.27, 95%CI 1.09 to 1.43) with placebo to reduce the risk of overall mortality compared to placebo. For AKI reduction, ns-MRAs (SUCRA 63.58%) have potential superiority. SGLT2is have comparable effects (RR 1.24, 95%CI 1.05 to 1.46) with placebo to reduce the risk of AKI. For hyperkalemia reduction, SGLT2is (SUCRA 93.12%) have potential superiority. SGLT2is have comparable effects (RR 1.24, 95%CI 1.05 to 1.46) with placebo to reduce the risk of AKI. Concerning hyperkalemia reduction, nsMRAs (RR 1.24 95%CI 0.39 to 3.72) and SGLT2is (RR 1.01 95%CI 0.40 to 3.02) did not show significant benefit compared to placebo.

**Conclusion:**

Concerning the efficacy and safety outcomes, SGLT2is may be recommended as a treatment regimen for maximizing kidney and cardiovascular protection, with a minimal risk of hyperkalemia in DKD.

**Systematic review registration:**

https://www.crd.york.ac.uk/prospero/, identifier CRD42023458613.

## 1 Introduction

Diabetes mellitus (DM) constitutes a significant threat to public health and presents a major healthcare burden in most countries. Preventive measures and appropriate management are indispensable to preventing long-term complications (1). As reported in the 10th Edition of the Diabetes Atlas by the International Diabetes Federation (IDF), the total number of DM patients (aged 20 to 79) worldwide has reached 537 million as of 2021, with an anticipated increase to 783 million by 2045 (2).

Sustained chronic hyperglycemia leads to vital damage both to macrovascular and microvascular systems, which are the main causes of cardiovascular and cerebrovascular diseases, peripheral artery disease, retinopathy, and nephropathy (3). Approximately 35% of patients with type 2 diabetes (T2D) are projected to develop chronic kidney disease (CKD). Diabetic kidney disease (DKD) is a critical complication of DM, and it is identified as a major cause of end-stage kidney disease (ESKD) (4). The characteristic of DKD is progressive proteinuria. Initially, the glomerular filtration rate (GFR) will gradually decline, resulting in failure eventually. The process is accompanied by capillary injury, mesangial cell proliferation, accumulation of extracellular matrix, thickening of the glomerular basement membrane, loss of podocytes, glomerulosclerosis, and ultimately renal tubulointerstitial fibrosis (5). DKD is a significant cause of ESKD and also serves as a critical risk factor for cardiovascular events and mortality (6). With the rapid rise in the prevalence of T2D worldwide and the significant number of patients developing DKD, there is a considerable economic burden (7). Therefore, it is crucial to slow down the progression through effective medical interventions, considering both a healthcare system and societal perspectives.

At present, the main therapies aimed at slowing down the progression of DKD involve blood pressure lowering, intensive glucose control, and blood lipid regulation, with angiotensin-converting enzyme inhibitors (ACEI) or angiotensin II receptor blockers (ARB) serving as the cornerstone of DKD management (8). The medications assist in mitigating the effects of the renin-angiotensin system, a key role in the onset and progression of DKD. The renoprotective effect of RAS blockade with ACEIs or ARBs is well established (9, 10). DKD patients have predominantly received treatment with RAS inhibitors, despite their limited efficacy, possibly as a result of angiotensin escape and/or aldosterone escape, which leads to an escalation in renin activity subsequent to the prolonged inhibition of renin-angiotensin-aldosterone system (11).

With the emergence of the new class of anti-hyperglycemia medications, ground breaking impulses were given to the treatment of DKD. Increasing evidence supports that employing new classes of medication, such as sodium-glucose cotransporter 2 (SGLT2is) and non-steroid mineralocorticoid receptor antagonist (ns-MRA) could potentially reduce the predefined composite renal outcomes and cardiovascular (CV) outcomes in patients with DKD. SGLT2is act by blocking the carrier protein SGLT2, which leads to glycosuria and subsequently initiates a cascade of positive effects on both the kidneys and cardiovascular system (12). ns-MRAs exhibit a high degree of specificity for mineralocorticoid receptor (MR). Consequently, this selective binding enables the nsMRAs to effectively counteract the effects of aldosterone, a steroid hormone that is believed to play a pivotal role in the progression of DKD (13).

Since there have been few trials directly comparing the efficacy and safety of SGLT2is and ns-MRAs, the debate continues regarding which interventions demonstrate the most significant renal and cardiovascular protective benefits. Network meta-analysis (NMA) serves as an extension of conventional meta-analysis. NMA enables the simultaneous comparison of multiple drug treatments for a disease, allowing for the decision of the most effective treatment option, in the absence of direct head-to-head evidence. Our study aims to identify the optimal treatment regimen, in conjunction with ACEI or ARB for patients with DKD. The study mainly centers around the following questions:

(1) Which medication should be administered to a patient with DKD to effectively prevent the progression of renal and cardiovascular composite outcomes?
(2) Which medication effectively decreases the risk of overall mortality, the development of end-stage kidney disease, acute kidney injury, and hyperkalemia?

To answer the questions, we performed a systemic review and NMA, to evaluate the efficacy and safety of SGLT2is and ns-MRAs for the treatment of DKD, and to provide an evidence-based basis for future clinical practice, medical decision-making, and research direction. Thus, the study provides an overview of the current state of DKD treatment and offers evidence-based guidance.

## 2 Methods

The study was carried out in line with the Preferred Reporting Items for Systematic Review and Meta-Analysis (PRISMA) 2020 statement (14) and was registered with PROSPERO (CRD 42023458613) https://www.crd.york.ac.uk/prospero/.

### 2.1 Data sources and search strategies

Database search was conducted using PubMed, Embase and Web of Science without any limitations on language from inception until January 20, 2024. The major search headings included “diabetic kidney disease”, “sodium glucose cotransporter 2 inhibitors” and “nonsteroidal mineralocorticoid receptor antagonists”. Mesh terms and free terms were searched (**Supplementary Table S1**).

### 2.2 Study selection and data extraction

Two reviewers (YSQ and WYH) independently screened the article titles, abstracts, and full texts to identify potential trials according to the following inclusion criteria:

(1) randomized controlled trials (RCTs) that compared SGLT2is or ns-MRAs with placebo or conventional treatment.
(2) DKD was defined as the presence of CKD in individuals with T2D. Adults (aged>18 years) with T2D and CKD. The clinical diagnosis of CKD is based on either of the following criteria:
  -Persistent high albuminuria defined as UACR≥30 mg/g but <300 mg/g and eGFR≥25 but <60 mL/min/1.73 m2 (Chronic Kidney Disease Epidemiology Collaboration);
  -Persistent very high albuminuria defined as UACR≥300 mg/g and eGFR≥25 but <75 mL/min/1.73 m2 (ChronicA Kidney Disease Epidemiology Collaboration).
(3) at least one of these outcomes must be included in the RCT: kidney-specific composite outcome; cardiovascular (CV)-specific composite outcome; ESKD; overall mortality.

Data extraction was conducted by two reviewers (YSQ and WYH), subsequently the members of the study (ZX, ZJ, LH) checked and eventually discussed the extracted data. The extracted information included trial name, registration number, study period, type of study, study drug, mean age, No. of male patients, sample size, outcome data.

### 2.3 Risk of bias and quality assessment

The Risk-of-Bias 2 tool (The Cochrane Collaboration’s tool for assessing risk of bias) will be used to assess and report the bias associated with the individual studies (15).

### 2.4 Outcomes

The efficacy outcomes assessed in the NMA include:

(1) kidney-specific composite outcome;
(2) CV-specific composite outcome;
(3) ESKD;
(4) overall mortality.

The kidney-specific composite outcome definitions differed among trials (**Supplementary Table S2**) but generally involved a combination of worsening eGFR or serum creatinine, ESKD with or without renal replacement therapy or transplant, or death from renal causes. The CV-specific composite outcome definitions varied across trials (**Supplementary Table S3**) but generally included a combination of CV death, nonfatal myocardial infarction (MI), nonfatal stroke or hospitalization for heart failure (HHF).

The safety outcomes assessed in the NMA include: hyperkalemia and acute kidney injury (AKI).

### 2.5 Statistical analysis calculated for each study to assess the effect sizes

The BUGSnet package in R (version 4.3.3) was utilized for statistical analysis of Bayesian network meta-analysis. Fixed- or random-effects models were selected for each outcome based on the deviance information criterion (DIC), using the model with the smallest value. Notably, a lower DIC value denotes a superior model fit in relation to the sample size. Risk ratios (RRs) was calculated to assess the effect sizes for dichotomous variables. All results are reported with 95% confidence intervals (CIs). The surface under the cumulative ranking curve (SUCRA) and mean ranks were used to rank the treatments for each outcome. A higher SUCRA value signifies that the treatment is more effective with respect to that outcome. As there were no closed loops, direct estimates could not be obtained in this review. Additionally, a P-value of less than 0.05 is indicative of statistical significance.

## 3 Results

### 3.1 Characteristics of included studies

**Figure 1:**
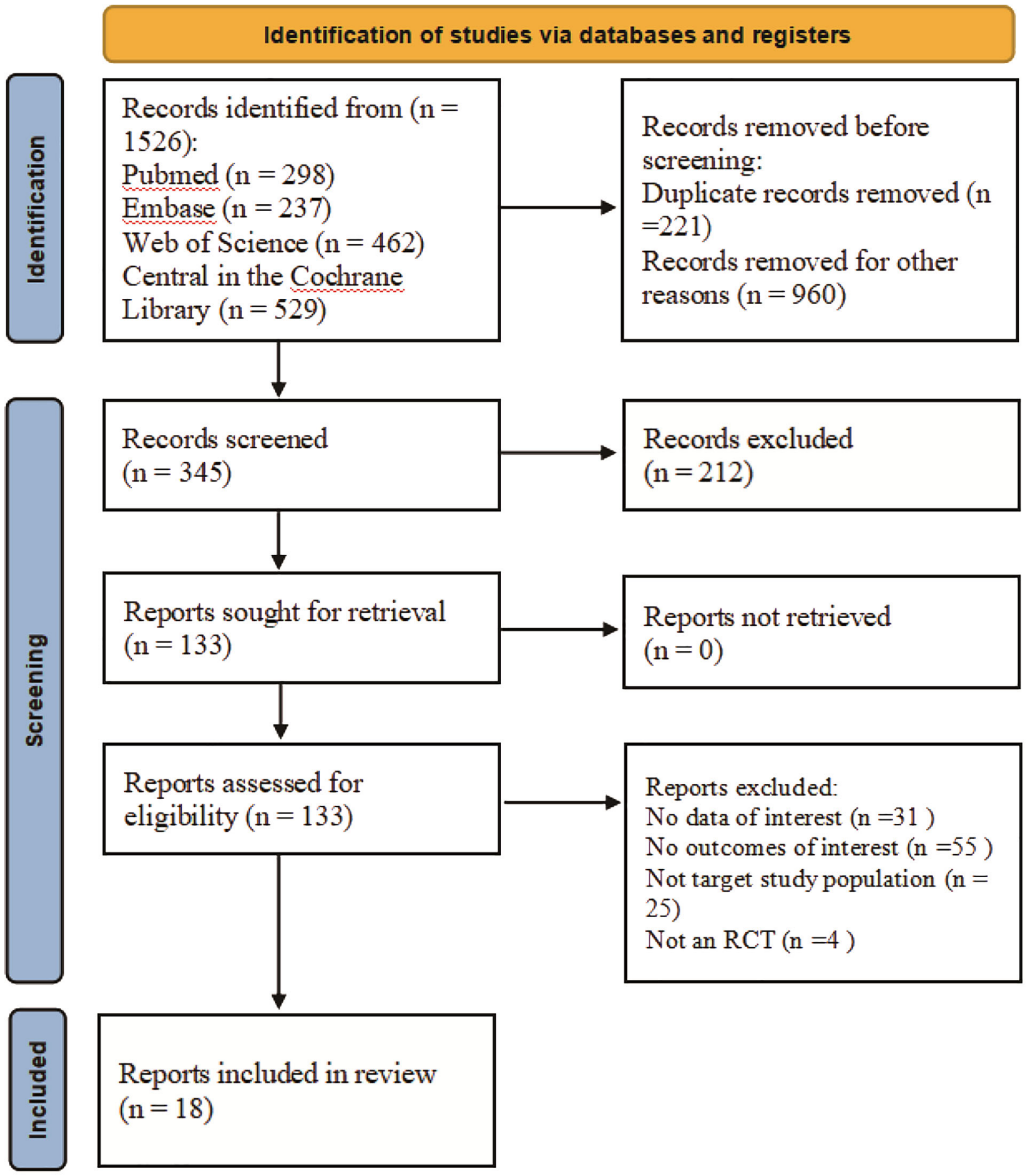
Flow diagram of the screening process.

The selection process was illustrated in a flowchart based on the PRISMA guidelines. **Figure 1** depicts the selection process. Our literature research initially yielded 1526 publications, out of which 10 RCTs (16–32) were found fully eligible, in total including 35,786 patients. The detailed characteristics of included studies are presented in **Table 1**. The main baseline information of included studies is summarized in **Table 2**. All RCTs had placebo as the comparator. The leverage plot was drawn for the option of fixed- or random-effects models (**Supplementary Figure S1**). The forest plot of NMA was drawn for the comparison for the efficacy and safety outcomes (**Supplementary Figure S2**).

**Table 1:** Characteristics of clinical trials included in the network meta-analysis.

Trial	Sponsor	Country	Follow-up, y	Study phase	Study drug	No. of patients (study group)	Age, y	Sex	Control drug	No. of patients (control group)	Age	Sex
DAPA-CKD	AstraZeneca	21	2.4	phase 3	Dapagliflozin	1455	64.1±9.8	961	placebo	1451	64.7±9.5	980
CREDENCE	Janssen	34	2.62(0.02, 4.53)	phase 3	Canagliflozin	2202	62.9±9.2	1440	placebo	2199	63.2±9.2	1467
CANVAS Program	Janssen	30	3.9	phase 3	Canagliflozin	1322	63.8±8.3	936	placebo	944	64.2±8.3	638
EMPA-REG OUTCOME	Boehringer Ingelheim	42	3.1	phase 3	Empagliflozin	1212	67.1±7.6	816	placebo	607	67.1±8.2	418
SCORED	Sanofi	44	1.18(0.86, 1.58)	phase 3	Sotagliflozin	5292	68.65±8.16	2945	placebo	5292	68.65±8.16	2885
ARTS-DN	Bayer	28	0.25	phase 2B	Finerenone	98	64.94±9.62	77	placebo	94	63.26±8.68	69
FIDELIO-DKD	Bayer	48	2.6	phase 3	Finerenone	2833	65.4±8.9	1953	placebo	2841	65.7±9.2	2030
JapicCTI173695	Daiichi-Sankyo	1	1.1	phase 2B	Esaxerenone	70	64±11	57	placebo	73	66±10	57
ESAX-DN	Daiichi-Sankyo	1	1.1	Phase 3	Esaxerenone	222	66±10	165	placebo	227	66±9	180
FIGARO-DKD	Bayer	48	3.4	phase 3	Finerenone	3686	64.1±9.7	2528	placebo	3666	64.1±10.0	2577

DAPA-CKD, Dapagliflozin and Prevention of Adverse Outcomes in Chronic Kidney Disease; CREDENCE, Canagliflozin and Renal Events in Diabetes with Established Nephropathy Clinical Evaluation; CANVAS, Canagliflozin Cardiovascular Assessment Study; EMPA‐REG OUTCOME, Empagliflozin, Cardiovascular Outcomes, and Mortality in Type 2 Diabetes; SCORED, Sotagliflozin Cardiovascular Outcomes Trial in Type 2 Diabetes Mellitus in Patients with Chronic Kidney Disease and Cardiovascular Disease; ARTS-DN, Advanced Renal Therapeutics Study of Diabetic Nephropathy; FIDELIO-DKD, Finerenone in Reducing Kidney Disease and Heart Failure Events in Patients with Type 2 Diabetes Mellitus and Chronic Kidney Disease; JapicCTI173695, Efficacy and Safety of Esaxerenone for the Treatment of Type 2 Diabetes with Microalbuminuria; ESAX-DN, Evaluation of the Effects of Esaxerenone on Renal and Cardiovascular Outcomes in Patients with Type 2 Diabetes Mellitus and Nephropathy; FIGARO-DKD, Finerenone in Reducing Kidney Failure and Disease Progression in Diabetic Kidney Disease; y, years. Sex indicates No. of male patients.

Mean ± SD; Median (interquartile range).

**Table 2:** Baseline information of clinical trials included in the network meta-analysis.

Trial	ClinicalTrials.gov number	UACR		eGFR		HbA1c		SBP	
		Study	Control	Study	Control	Study	Control	Study	Control
DAPA-CKD	NCT03036150	#1024.5(472.5-2111.0)	#1004.5(493.3-2017.0)	44.0±12.6	43.6±12.6	7.8±1.7	7.8±1.6	138.8±17.6	139.6±17.1
CREDENCE	NCT02065791	#923(459–1794)	#931(473–1868)	56.3±18.2	56.0±18.3	8.3±1.3	8.3±1.3	139.8±15.6	140.2±15.6
CANVAS Program	NCT01032639	#67.1 (42.6–127.2)	#69.4 (44.6–120.5)	74.8±20.9	73.9±21.9	8.4±1.0	8.4±0.9	139.4±15.6	139.2±16.2
EMPA-REG OUTCOME	NCT01131676	N	N	48.4±8.2	48.6±7.8	8.07±0.86	8.03±0.85	136.1±18.0	136.4±18.7
SCORED	NCT03315143	#74 (18–486)	#75 (17–477)	#44.4 (37.0–51.3)	#44.7 (37.0–51.5)	#8.3 (7.6–9.3)	#8.3 (7.6–9.4)	#138 (127–149)	#139 (127–149)
ARTS-DN	NCT1874431	$249.5 (30.4-3917)	$188.4 (15.0-3056)	67.0±20.9	72.2±20.4	7.7±1.2	7.6±1.3	137.6±14.0	139.9±14.3
FIDELIO-DKD	NCT02540993	N	N	N	N	7.7±1.3	7.7±1.4	138.1±14.3	138.0±14.4
JapicCTI173695	NCT02345057	▴112 (100-126)	▴110 (98-123)	68±19	69±19	7.0±0.6	6.9±0.6	137±13	138±11
ESAX-DN	NCT02345057	$113 (46, 286)	$110 (47, 278)	69±18	69±18	7.0±0.6	7.0±0.6	140±10	140±10
FIGARO-DKD	NCT02545049	#302 (105–749)	#315 (111–731)	67.6±21.7	68.0±21.7	7.7±1.4	7.7±1.4	135.8±14.0	135.7±14.1

DAPA-CKD, Dapagliflozin and Prevention of Adverse Outcomes in Chronic Kidney Disease; CREDENCE, Canagliflozin and Renal Events in Diabetes with Established Nephropathy Clinical Evaluation; CANVAS, Canagliflozin Cardiovascular Assessment Study; EMPA‐REG OUTCOME, Empagliflozin, Cardiovascular Outcomes, and Mortality in Type 2 Diabetes; SCORED, Sotagliflozin Cardiovascular Outcomes Trial in Type 2 Diabetes Mellitus in Patients with Chronic Kidney Disease and Cardiovascular Disease; ARTS-DN, Advanced Renal Therapeutics Study of Diabetic Nephropathy; FIDELIO-DKD, Finerenone in Reducing Kidney Disease and Heart Failure Events in Patients with Type 2 Diabetes Mellitus and Chronic Kidney Disease; JapicCTI173695, Efficacy and Safety of Esaxerenone for the Treatment of Type 2 Diabetes with Microalbuminuria; ESAX-DN, Evaluation of the Effects of Esaxerenone on Renal and Cardiovascular Outcomes in Patients with Type 2 Diabetes Mellitus and Nephropathy; FIGARO-DKD, Finerenone in Reducing Kidney Failure and Disease Progression in Diabetic Kidney Disease; UACR, Urine albumin-creatinine ratio; eGFR, Estimated glomerular filtration rate; HbA1c, Hemoglobin glycation; SBP, systolic blood pressure.

Mean ± SD; #Median (interquartile range); $Median (min, max); ▴90%CI.

### 3.2 Risk of bias in included studies

**Figure 2:**
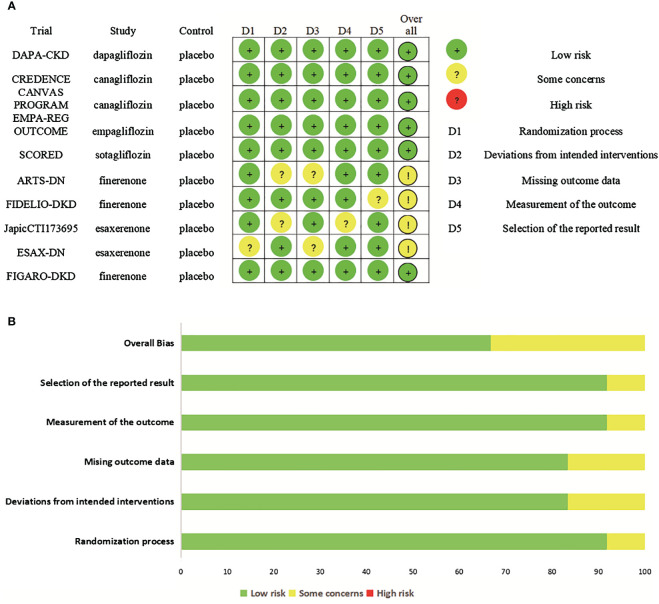
Risk of bias. **(A)** Risk of bias summary for included studies, showing each risk of bias item for every included study. **(B)** Risk of bias graph presenting each risk of bias item as percentages across all included studies.

Six RCTs had an overall low risk of bias. Four RCTs had some concerns. Two RCTs had some concerns for deviations from intended interventions (17, 21), two for missing outcome data (17, 26), one for randomization process (26), one for measurement of the outcome (21), one for selection of the reported result (24). The risk of bias assessments of included studies are shown in **Figure 2**.

### 3.3 Efficacy outcomes

**Figure 3:**
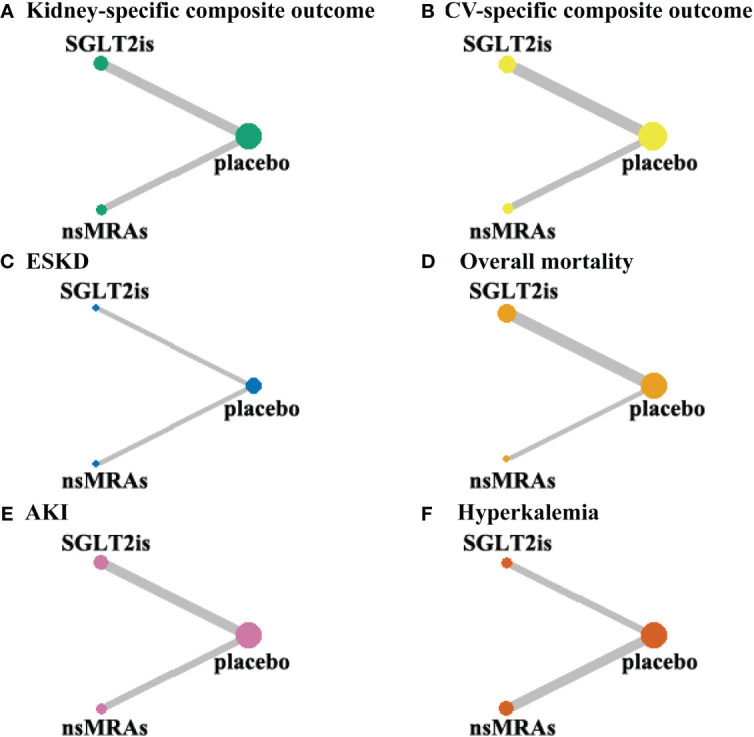
Evidence network maps. **(A-F) **were drawn evidence network plots of different treatments for DKD with kidney-specific composite outcome, CV-specific composite outcome, ESKD, overall mortality, AKI, and hyperkalemia as outcomes, respectively. The thickness of line segments in the figure represented the number of studies, that could be directly compared between the two connected interventions, and the size of the points represented the total sample size of the intervention included in the study. SGLT2is, sodium glucose cotransporter 2 inhibitors; nsMRAs, non steroid mineralocorticoid receptor antagonists; CV, cardiovascular; ESKD, end-stage kidney disease; AKI, acute kidney disease.

**Figure 3** shows the network of treatment comparisons in available trials. In this network meta-analysis, none of the six outcomes had a closed loop. This implies that neither outcomes from direct nor indirect comparisons are considered, which renders the need for assessing coherence redundant.

#### 3.3.1 Kidney-specific composite outcome

The analyses of kidney-specific composite outcome included six studies (four with SGLT2is, two with ns-MRAs), that had enrolled a total of 33,183 participants.

**Figure 4:**
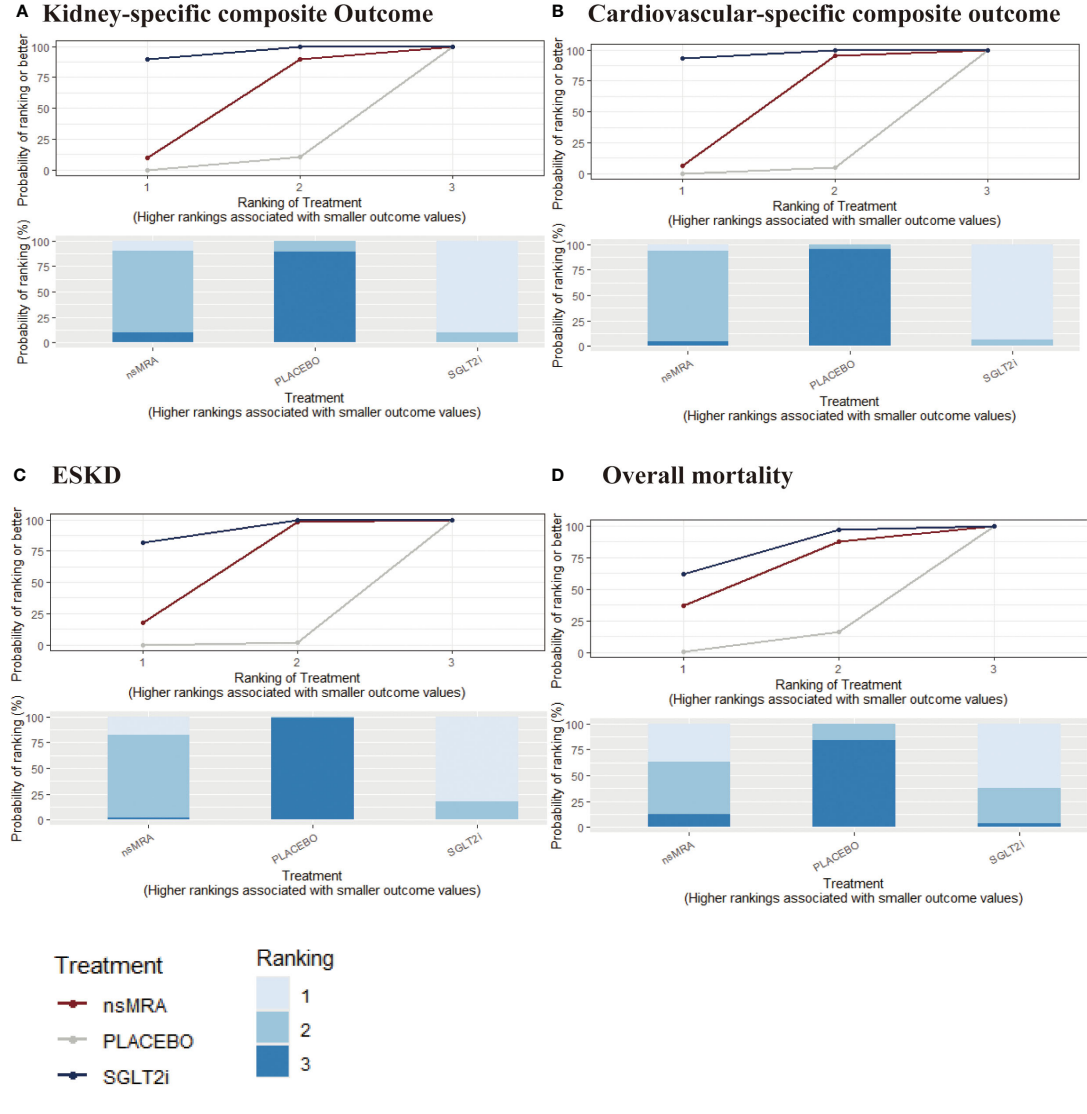
SUCRA probabilities for the effectiveness outcomes of interventions. **(A-D)** were drawn SUCRA probabilities plots for the effectiveness outcomes of interventions. **(A)** kidney-specific composite outcome; **(B)** CV-specific composite outcome; **(C)** ESKD; **(D)** overall mortality. A higher SUCRA indicates a lower probability that the drug can reach the endpoint. SGLT2i, sodium glucose cotransporter 2 inhibitor; nsMRA, non steroid mineralocorticoid receptor antagonist; ESKD, end-stage kidney disease.

**Figure 5:**
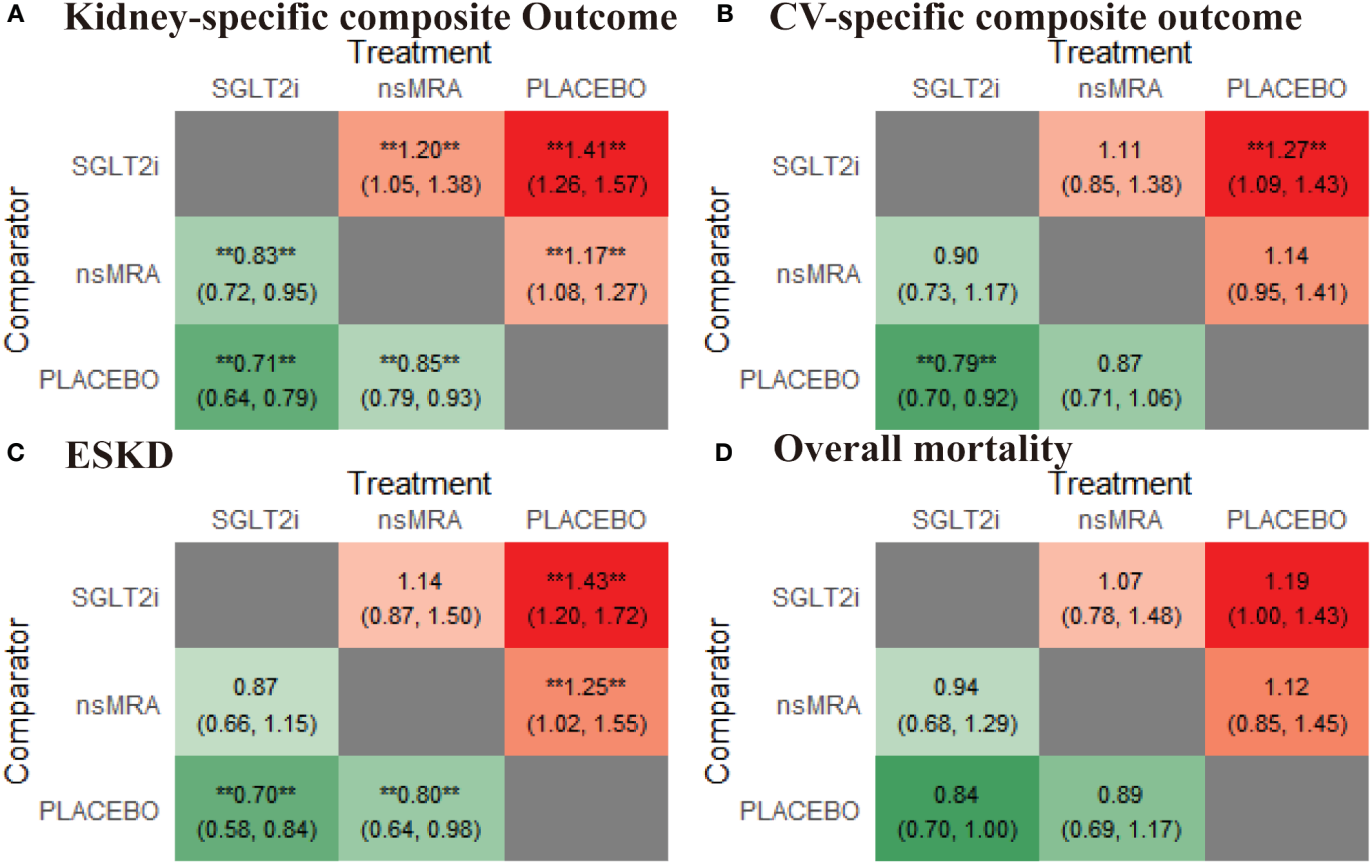
Comparative heatmap for effectiveness outcomes of interventions. **(A-D)** drawn comparative heat map of interventions with the kidney-specific composite outcome, CV-specific composite outcome, ESKD, overall mortality. Comparisons between treatments should be read from left to right. The effect size (RR) and 95% confidence interval are in the cell in common between the column-defining treatment and the row-defining treatment. RR>1 favors the row-defining treatment. SGLT2i, sodium glucose cotransporter 2 inhibitor; nsMRA, non steroid mineralocorticoid receptor antagonist; CV, Cardiovascular; ESKD, end-stage kidney disease; RR, Risk ratio. **represents statistical significance.

The rankogram (**Figure 4A**) showed that SGLT2is had the superior probability of reducing the risk of kidney-specific composite outcome (99.84%), followed by ns-MRAs (50.15%). For kidney-specific composite outcome reduction, SGLT2is were most likely to rank best. When compared with placebo, both SGLT2is (RR 1.41, 95% CI 1.26 to 1.57) and ns-MRAs (RR 1.17, 95% CI 1.08 to 1.27) were associated with reductions in kidney-specific composite outcome (**Figure 5A**). SGLT2is were associated with a lower risk of kidney-specific composite outcome compared with ns-MRAs (RR 1.20, 95% CI 1.05 to 1.38).

#### 3.3.2 CV-specific composite outcome

The analyses of CV-specific composite outcome included eight studies (five with SGLT2is, three with ns-MRAs), that had enrolled a total of 35,194 participants.

The rankogram (**Figure 4B**) showed that SGLT2is had the superior probability of reducing the risk of CV-specific composite outcome (91.61%), followed by ns-MRAs (55.17%). For CV-specific composite outcome reduction, SGLT2is were most likely to rank best. Compared with the placebo, SGLT2is (RR 1.27, 95% CI 1.09 to 1.43) were associated with reductions in CV-specific composite outcome significantly (**Figure 5B**). And ns-MRAs (RR 1.14, 95% CI 0.95 to 1.41) were similar in CV-specific composite outcome reduction compared with placebo.

#### 3.3.3 ESKD

The analyses of ESKD included four studies (two with SGLT2is, two with ns-MRAs), that had enrolled a total of 20,333 participants.

The rankogram (**Figure 4C**) showed that SGLT2is had the superior probability of reducing the risk of ESKD (91.38%), followed by ns-MRAs (57.81%). For ESKD, SGLT2is were most likely to rank best. When compared with placebo, both SGLT2is (RR 1.43, 95% CI 1.20 to 1.72) and ns-MRAs (RR 1.25, 95% CI 1.02 to 1.55) were associated with reductions in ESKD (**Figure 5C**).

#### 3.3.4 Overall mortality

The analyses of overall mortality included nine studies (five with SGLT2is, four with ns-MRAs), that had enrolled a total of 35,594 participants.

The rankogram (**Figure 4D**) showed that SGLT2is had the superior probability of reducing the risk of overall mortality (83.03%), followed by ns-MRAs (57.5%). For overall mortality, SGLT2is were most likely to rank best. We found that treatments were similar in overall mortality reduction since compared with placebo, neither of the drugs showed significant changes [SGLT2is (RR 1.19, 95% CI 1.00 to 1.43), ns-MRAs (RR 1.12, 95% CI 0.85 to 1.45)] (**Figure 5D**).

### 3.4 Safety outcomes

#### 3.4.1 AKI

The analyses of hyperkalemia included seven studies (four with SGLT2is, three with ns-MRAs), that had enrolled a total of 24,610 participants.

**Figure 6:**
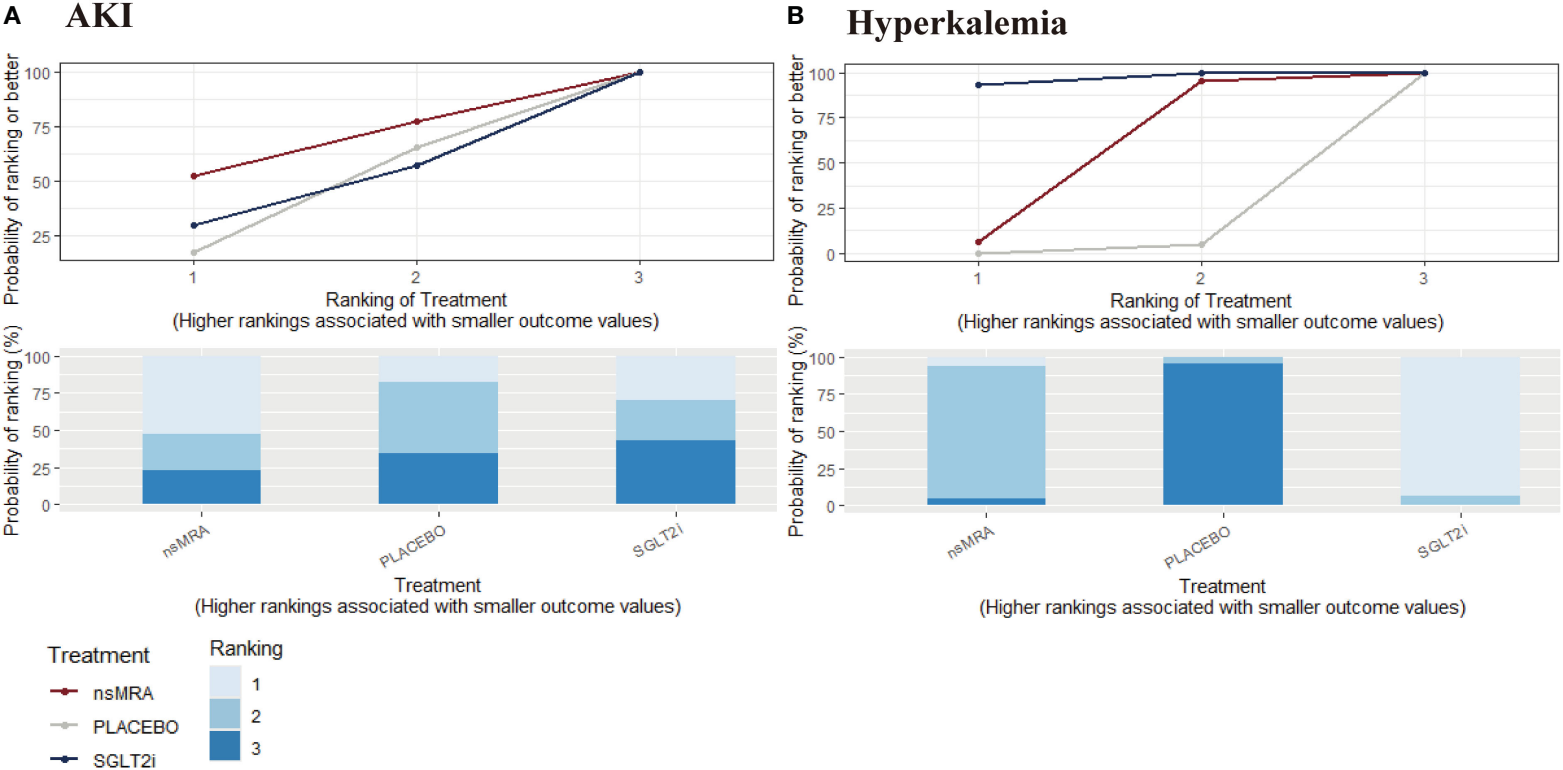
SUCRA probabilities for safety outcomes of interventions. **(A, B)** were drawn SUCRA probabilities plots for the safety outcomes of interventions. **(A)** AKI; **(B)** Hyperkalemia. A higher SUCRA indicates a lower probability that the drug can reach the endpoint. SGLT2i, sodium glucose cotransporter 2 inhibitor; nsMRA, non steroid mineralocorticoid receptor antagonist; AKI, acute kidney disease.

**Figure 7:**
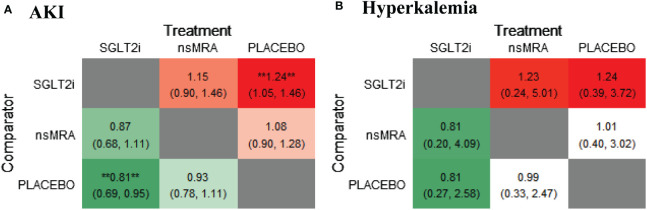
Comparative heatmap for safety outcomes of interventions. **(A, B)** drawn comparative heat map of interventions with AKI and hyperkalemia. Comparisons between treatments should be read from left to right. The effect size (RR) and 95% confidence interval are in the cell in common between the column-defining treatment and the row-defining treatment. RR>1 favors the row-defining treatment. SGLT2i, sodium glucose cotransporter 2 inhibitor; nsMRA, non steroid mineralocorticoid receptor antagonist; AKI, Acute kidney injury; RR, Risk ratio. **represents statistical significance.

The rankogram (**Figure 6A**) showed that ns-MRAs had the superior probability of reducing the risk of AKI (63.58%), followed by SGLT2is (44.76%). For AKI reduction, ns-MRAs were most likely to rank best. Compared with the placebo, SGLT2is (RR 1.24, 95% CI 1.05 to 1.46) were associated with reductions in AKI significantly (**Figure 7A**). And ns-MRAs (RR 1.08, 95% CI 0.90 to 1.28) were similar in AKI reduction compared with placebo.

#### 3.4.2 Hyperkalemia

The analyses of hyperkalemia included nine studies (four with SGLT2is, five with ns-MRAs), that had enrolled a total of 32, 880 participants.

The rankogram (**Figure 6B**) showed that SGLT2is had the superior probability of reducing the risk of hyperkalemia (93.12%), followed by ns-MRAs (46.18%). For hyperkalemia reduction, SGLT2is were most likely to rank best. We found that treatments were similar in hyperkalemia reduction since compared with placebo, neither of the drugs showed significant changes [SGLT2is (RR 1.24, 95% CI 0.39 to 3.72), ns-MRAs (RR 1.01, 95% CI 0.40 to 3.02)] (**Figure 7B**).

## 4 Discussion

This Bayesian NMA included 10 RCTs, encompassing 35,786 participants. The efficacy and safety outcomes in the study were mainly derived from the DAPA-CKD, CREDENCE, CANVAS Program, EMPA-REG OUTCOME, SCORED, ARTS-DN, FIDELIO-DKD, JapicCTI173695, ESAX-DN, and FIGARO-DKD trials. The main comparison subjects were ns-MRAs (finerenone or esaxerenone) and SGLT2is (canagliflozin, dapagliflozin, empagliflozin or sotagliflozin.

The study compared SGLT2is and ns-MRAs to provide comprehensive evidence of various drug regimens in the DKD population for improving kidney and cardiovascular prognosis. The primary strength of the NMA lies in its targeted focus on DKD patients. This specialized emphasis has facilitated, for the first time, a comparison of the efficacy and safety of innovative antidiabetic drug classes in relation to their impacts on cardiorenal outcomes, specifically within DKD patients.

The findings of the study revealed that, when compared to ns-MRAs, the utilization of SGLT2is resulted in the superior favorable outcomes. SGLT2is have been demonstrated a significant reduction in the risks associated with kidney-specific composite outcome, CV-specific composite outcome, ESKD, overall mortality by 41%, 27%, 43% and 19%, respectively, compared with placebo. While ns-MRAs lowered the risks of kidney-specific composite outcome and ESKD by 17% and 25%, respectively, there was no benefit with regard to the risk of CV-specific composite outcome and overall mortality compared with placebo. As for safety outcomes, the NMA findings suggest that the use of SGLT2is was linked to reduced hyperkalemia; rather, the use of ns-MRAs was associated with reduced AKI. Overall, SGLT2is may be favored over ns-MRAs based on their significant correlation with lower mortality and their additional cardiorenal benefits. SGLT2is ought to be considered an essential addition to the treatment regimen for each patient diagnosed with DKD.

### 4.1 The potential organ-protective effects of SGLT2is and MRAs

Clinical studies have demonstrated that SGLT2is not only lower blood glucose levels but also have the ability to decrease blood pressure and postpone the onset and/or progression of kidney dysfunction (23, 25, 33). The Kidney Disease: Improving Global Outcomes (KDIGO) diabetes workgroup recommends SGLT2is as a first-line treatment for patients with T2DM who also have CKD (34). The SGLT2is exerts its therapeutic effects by blocking the reabsorption of filtered sodium ions and glucose within the proximal tubule (35). This inhibition increases sodium ion transportation, and the subsequent rise in sodium concentration prompts activation of the tubuloglomerular feedback at the macula densa, which triggers constriction of the afferent arteriole (36). This constriction induces a decline in plasma flow and intra-glomerular capillary pressure, thereby culminating in the reduction of glomerular filtration rate (GFR) observed following the intake of SGLT2is (37).

Patients with T2D are exposed to substantial risks of mortality and the probability of developing complications due to unresolved metabolic, cellular, and inflammatory events (38). Upregulation of the MR has been detected in an assortment of clinical conditions, including hyperglycemia, obesity, insulin resistance, high salt intake, and CKD (39–43). Under pathological conditions, the overactivation of the MR prompts the increase of diverse proinflammatory and profibrotic cytokines (e.g., tumor necrosis factor receptor-α, interleukin-1, fibronectin, and transforming growth factor-α) within the cardiovascular and renal system, ultimately resulting in chronic inflammation and fibrosis (6, 44). Accordingly, MR antagonists have certain benefits in lowering urinary albumin, inhibiting tissue fibrosis, and offering cardiorenal protective effect (44, 45). Traditional MR antagonists, including spironolactone and eplerenone, are not widely used clinically owing to the high incidence of hyperkalemia and gynecomastia (46). Traditional MR antagonists are generally used as diuretics in the management of hypertension, primary aldosteronism, and heart failure (47). Over the past decade, novel and first-in-class ns-MRAs (finerenone, esaxerenone) have been developed and differ significantly with traditional MR antagonists due to their higher selective MRA activity, devoid of androgen antagonism and progesterone agonism (48). The U.S. Food and Drug Administration has granted approval for finerenone to reduce the risks of decline in kidney function, kidney failure, cardiovascular death, nonfatal myocardial infarctions, and hospitalization for heart failure in adults with CKD associated with T2D (49).

### 4.2 Advantage of the use of SGLT2is in DKD patients

Based on the available evidence of our study, SGLT2is had the highest SUCRA rank for all efficacy outcomes, indicating that they possess a greater probability than ns-MRAs of reducing risks of both CV and renal outcomes. Obesity is considered to be closely related to insulin resistance. The weight-reducing effect of SGLT2is could improve insulin resistance, regulate energy homeostasis, and thereby exert renal protective effects that go beyond glucose-lowering (50). It has been proposed that SGLT2is maintain the balance of sodium-potassium ion exchange (natriuretic and diuretic effects) and energy metabolism, as well as reduce oxidative stress in cells to provide favorable effects on the risk of renal protection (51, 52). A study by Ravindran et al. (53) suggested that SGLT2is are capable of inhibiting the RAAS activation, playing a role in lowering blood pressure and improving renal hyperfiltration.

### 4.3 Risks associated with the use of ns-MRAs

Adverse events associated with ns-MRAs are a common concern in clinical practice. For ns-MRAs, some common issues include hyperkalemia, upper respiratory tract infections, pharyngitis, abdominal discomfort, constipation, dizziness, and renal impairment. Among various adverse events, hyperkalemia has always been the major concerning, as the use of ns-MRAs could lead to decreased potassium excretion due to the blockade of aldosterone receptors, resulting in an elevated level of serum potassium (54). Although the incidence of hyperkalemia has increased compared to the placebo, there is study showing that the risk of ns-MRAs is lower than that of traditional steroidal MRA (55). In addition, as some trials included a combination of RAS inhibitors, there is a possibility of increased blood potassium changes. Studies have found that serum potassium elevation most commonly occurs in subsets with low baseline eGFR or high baseline potassium levels. It has been observed that there tends to be a generally stable return to baseline blood potassium levels by the final stages of treatment, or upon treatment completion (56, 57).

### 4.4 The related meta-analysis

In a comprehensive meta-analysis (58), it was demonstrated that the utilization of SGLT2is can lead to a significant reduction in glycated hemoglobin, blood pressure, body weight, and proteinuria levels. Furthermore, these outcomes have been shown to postpone the decline in the eGFR (with a significant difference when compared to placebo: 1.35 95% CI: 0.78-1.93). Additionally, the study revealed that the risks of renal composite outcome, encompassing the doubling of serum creatinine levels, ESKD, or renal death, were considerably reduced in patients treated with SGLT2is (RR 0.71, 95% CI: 0.53-0.95). Compared to patients who did not use SGLT2is, those who did see a delayed decline in GFR slope. This conclusion also effectively explains the renal protection of SGLT2is.

A NMA (59) based on 18 large trials comparing SGLT2i, finerenone, and glucagonlike peptide-1 receptor agonists (GLP-1 RA) found finerenone can decrease the risk of major adverse cardiovascular events, renal outcome, and HHF, along with the tendency to reduce all-cause death in patients with T2DM and CKD. GLP-1 RA showed a marked impact on reducing cardiovascular events when juxtaposed with exendin-4 analogues. The study further elucidated that finerenone boasts the same potency as SGLT2is in lowering MACE risk. The inconsistency observed may be credited to the different definition of CV- specific composite outcome, specifically the inclusion of unstable angina, or without trials of ESAX-DN.

A comprehensive NMA (60) comprising data from 42346 patients with DKD, had compared all treatments for DKD that have been used by nephrologists over the last two decades. The study found SGLT2is should be a crucial part of the treatment of DKD patients alongside either ACEI or ARB, concerning mortality and ESKD. The finding aligns with the results observed in our study.

### 4.5 Limitations

Our study has certain limitations that warrant further discussion. First, our search was confined to placebo-controlled trials only. To date, there have been no RCTs that focus on kidney or cardiovascular outcomes to directly compare the efficacy of SGLT2is and ns-MRAs in patients diagnosed with DKD. Additionally, the absence of closed-loop meta-analyses resulted in findings from our network meta-analysis producing limited insights.

Second, it is of note that FIDELIO-DKD and FIGARO-DKD trials permitted the concurrent administration of finerenone in conjunction with SGLT2is (with 6.4% of patients receiving SGLT2is at baseline), whereas combination therapy with SGLT2is and ns-MRA was limited in CREDENCE and DAPA-CKD trials (as baseline ns-MRA use was prohibited). This could potentially influence the difference in renal and cardiac outcomes between the two agents.

Third, the follow-up period for the trials included varied, ranging from 12 to 104 weeks. Therefore, there may be some differences in kidney-specific composite outcome in patients with DKD in this study.

Finally, there were notable differences in baseline levels of HbA1c and eGFR between patients receiving SGLT2is and ns-MRA treatments. The absence of pooled individual patient data and the small number of eligible trials restricted our capacity to conduct substantial subgroup analyses based on factors like the stage of DKD, the presence of albuminuria. Additionally, since most trials included in this meta-analysis excluded participants with eGFR<25 ml/min/1.73 m^2^, our findings may not be applicable to patients with severe renal insufficiency. Future studies are required to clarify the effectiveness of ns-MRAs and SGLT2is in individuals with diverse clinical profiles.

## 5 Conclusion

The latest Standards of Medical Care in Diabetes guidelines from the American Diabetes Association (ADA) recommend the use of SGLT2is in patients with stage 3 CKD or higher and T2DM regardless of glycemic control, to slow the progression of CKD and reduce CV and renal risks. Our data lend further support to ADA and KDIGO guidelines, which suggest that SGLT2is are needed in DKD.

## Data availability statement

The original contributions presented in the study are included in the article/**Supplementary Material**. Further inquiries can be directed to the corresponding authors.

## Author contributions

S-QY: Data curation, Methodology, Visualization, Writing – original draft. XZ: Conceptualization, Funding acquisition, Supervision, Writing – review & editing. JZ: Data curation, Writing – review & editing. HL: Data curation, Writing – review & editing. Y-HW: Data curation, Methodology, Writing – review & editing. Y-GW: Funding acquisition, Writing – review & editing.
