# Supplementary material for: Comparative efficacy and safety of SGLT2is and ns-MRAs in patients with diabetic kidney disease: a systematic review and network meta-analysis
Source: Front Endocrinol (Lausanne). 2024 Jul 4;15:1429261. doi: 10.3389/fendo.2024.1429261 (PMC11256196; doi:10.3389/fendo.2024.1429261)
Supplement: Supplementary file 1 [file DataSheet_1.docx]

| **Our search strategy is as follows, taking it in Pubmed for example:** |
| --- |
| Diabetic[Title/Abstract]) OR (Nephropathy, Diabetic[Title/Abstract])) OR (Diabetic Nephropathy[Title/Abstract])) OR (Diabetic Kidney Disease[Title/Abstract])) OR (Diabetic Kidney Diseases[Title/Abstract])) OR (Kidney Disease, Diabetic[Title/Abstract])) OR (Kidney Diseases, Diabetic[Title/Abstract])) OR (Glomerulosclerosis, Diabetic[Title/Abstract])) OR (Intracapillary Glomerulosclerosis[Title/Abstract])) OR (Diabetic Glomerulosclerosis[Title/Abstract])) OR (Nodular Glomerulosclerosis[Title/Abstract])) OR (Glomerulosclerosis, Nodular[Title/Abstract])) OR (Kimmelstiel-Wilson Syndrome[Title/Abstract])) OR (Kimmelstiel Wilson Syndrome[Title/Abstract])) OR (Syndrome, Kimmelstiel-Wilson[Title/Abstract])) OR (Kimmelstiel-Wilson Disease[Title/Abstract])) OR (Kimmelstiel Wilson Disease[Title/Abstract]))) AND ((("Mineralocorticoid Receptor Antagonists"[Mesh]) OR ((((((((((((((((((((((Receptor Antagonists, Mineralocorticoid[Title/Abstract]) OR (Antagonists, Mineralocorticoid Receptor[Title/Abstract])) OR (Mineralocorticoid Antagonists[Title/Abstract])) OR (Antagonists, Mineralocorticoid[Title/Abstract])) OR (Aldosterone Receptor Antagonist[Title/Abstract])) OR (Antagonist, Aldosterone Receptor[Title/Abstract])) OR (Receptor Antagonist, Aldosterone[Title/Abstract])) OR (Mineralocorticoid Antagonist[Title/Abstract])) OR (Antagonist, Mineralocorticoid[Title/Abstract])) OR (Mineralocorticoid Receptor Antagonist[Title/Abstract])) OR (Antagonist, Mineralocorticoid Receptor[Title/Abstract])) OR (Receptor Antagonist, Mineralocorticoid[Title/Abstract])) OR (Aldosterone Receptor Antagonists[Title/Abstract])) OR (Antagonists, Aldosterone Receptor[Title/Abstract])) OR (Receptor Antagonists, Aldosterone[Title/Abstract])) OR (Aldosterone Antagonists[Title/Abstract])) OR (Antagonists, Aldosterone[Title/Abstract])) OR (Aldosterone Antagonist[Title/Abstract])) OR (Antagonist, Aldosterone[Title/Abstract])) OR (Eplerenone[Title/Abstract])) OR (Canrenone[Title/Abstract])) OR (Finerenone[Title/Abstract]))) OR (("Sodium-Glucose Transporter 2 Inhibitors"[Mesh]) OR ((((((((((((((((((((Sodium Glucose Transporter 2 Inhibitors[Title/Abstract]) OR (SGLT-2 Inhibitors[Title/Abstract])) OR (SGLT 2 Inhibitors[Title/Abstract])) OR (SGLT2 Inhibitors[Title/Abstract])) OR (Sodium-Glucose Transporter 2 Inhibitor[Title/Abstract])) OR (Sodium Glucose Transporter 2 Inhibitor[Title/Abstract])) OR (SGLT2 Inhibitor[Title/Abstract])) OR (Inhibitor, SGLT2[Title/Abstract])) OR (Gliflozins[Title/Abstract])) OR (Gliflozin[Title/Abstract])) OR (SGLT-2 Inhibitor[Title/Abstract])) OR (Inhibitor, SGLT-2[Title/Abstract])) OR (SGLT 2 Inhibitor[Title/Abstract])) OR (Dapagliflozin[Title/Abstract])) OR (Canagliflozin[Title/Abstract])) OR (Luseogliflozin[Title/Abstract])) OR (Sotagliflozin[Title/Abstract])) OR (Ertugliflozin[Title/Abstract])) OR (Ipragliflozin[Title/Abstract])) OR (Tofogliflozin[Title/Abstract]))))) AND (randomized controlled trial[Publication Type] OR randomized[Title/Abstract] OR placebo[Title/Abstract]) |
