# Supplementary material for: Comparative efficacy and safety of SGLT2is and ns-MRAs in patients with diabetic kidney disease: a systematic review and network meta-analysis
Source: Front Endocrinol (Lausanne). 2024 Jul 4;15:1429261. doi: 10.3389/fendo.2024.1429261 (PMC11256196; doi:10.3389/fendo.2024.1429261)
Supplement: Supplementary file 2 [file DataSheet_2.docx]

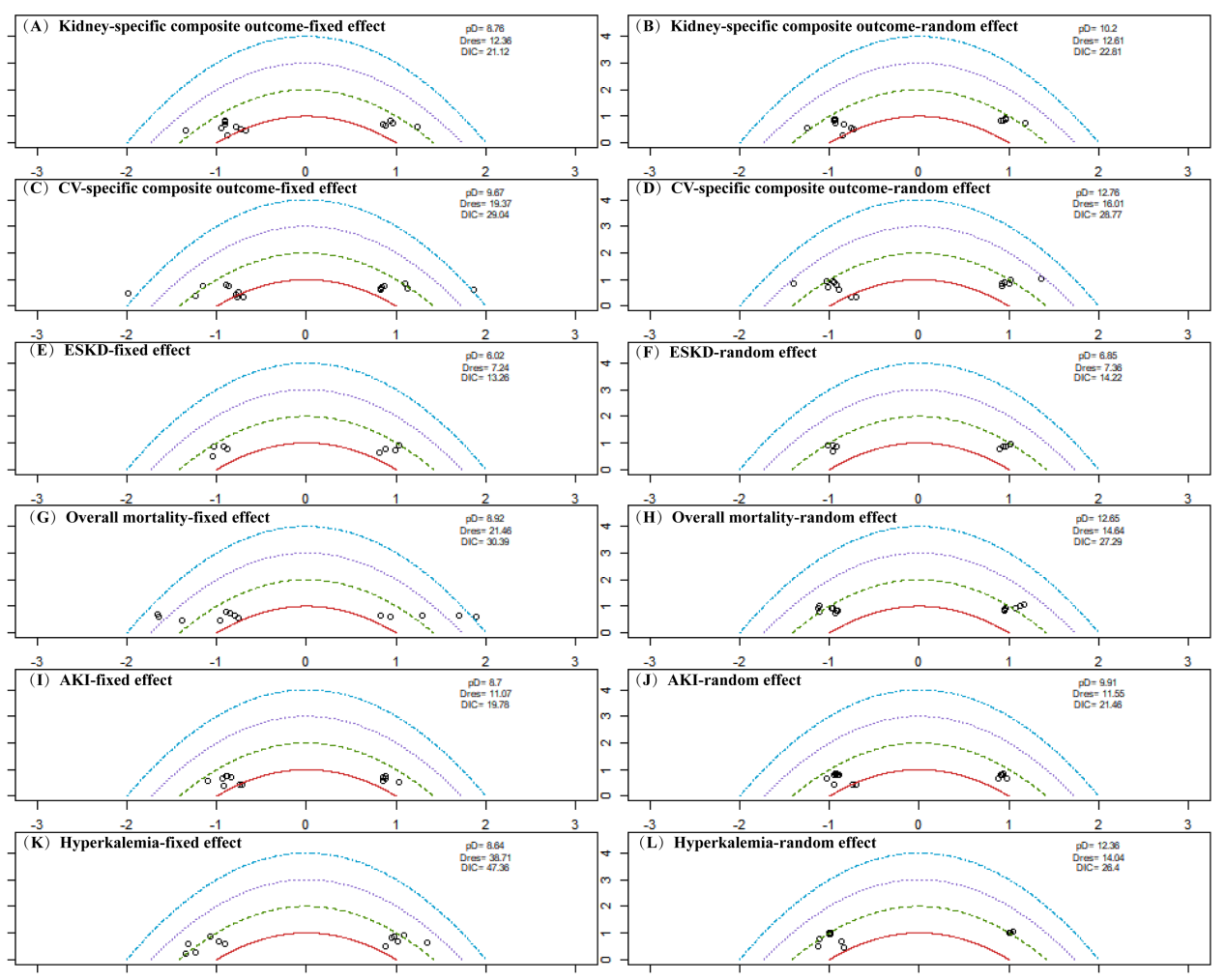


**Supplement Figure 1.|** A-L drawn leverage plot for the option of fixed- or random-effects models. Fixed- or random-effects models were selected for each outcome based on the DIC, using the model with the smallest value. Notably, a lower DIC value denotes a superior model fit in relation to the sample size. CV, Cardiovascular; ESKD, End-stage kidney disease; AKI, Acute kidney injury; DIC, Deviance information criterion.
