# Supplementary material for: Comparative efficacy and safety of SGLT2is and ns-MRAs in patients with diabetic kidney disease: a systematic review and network meta-analysis
Source: Front Endocrinol (Lausanne). 2024 Jul 4;15:1429261. doi: 10.3389/fendo.2024.1429261 (PMC11256196; doi:10.3389/fendo.2024.1429261)
Supplement: Supplementary file 3 [file DataSheet_3.docx]

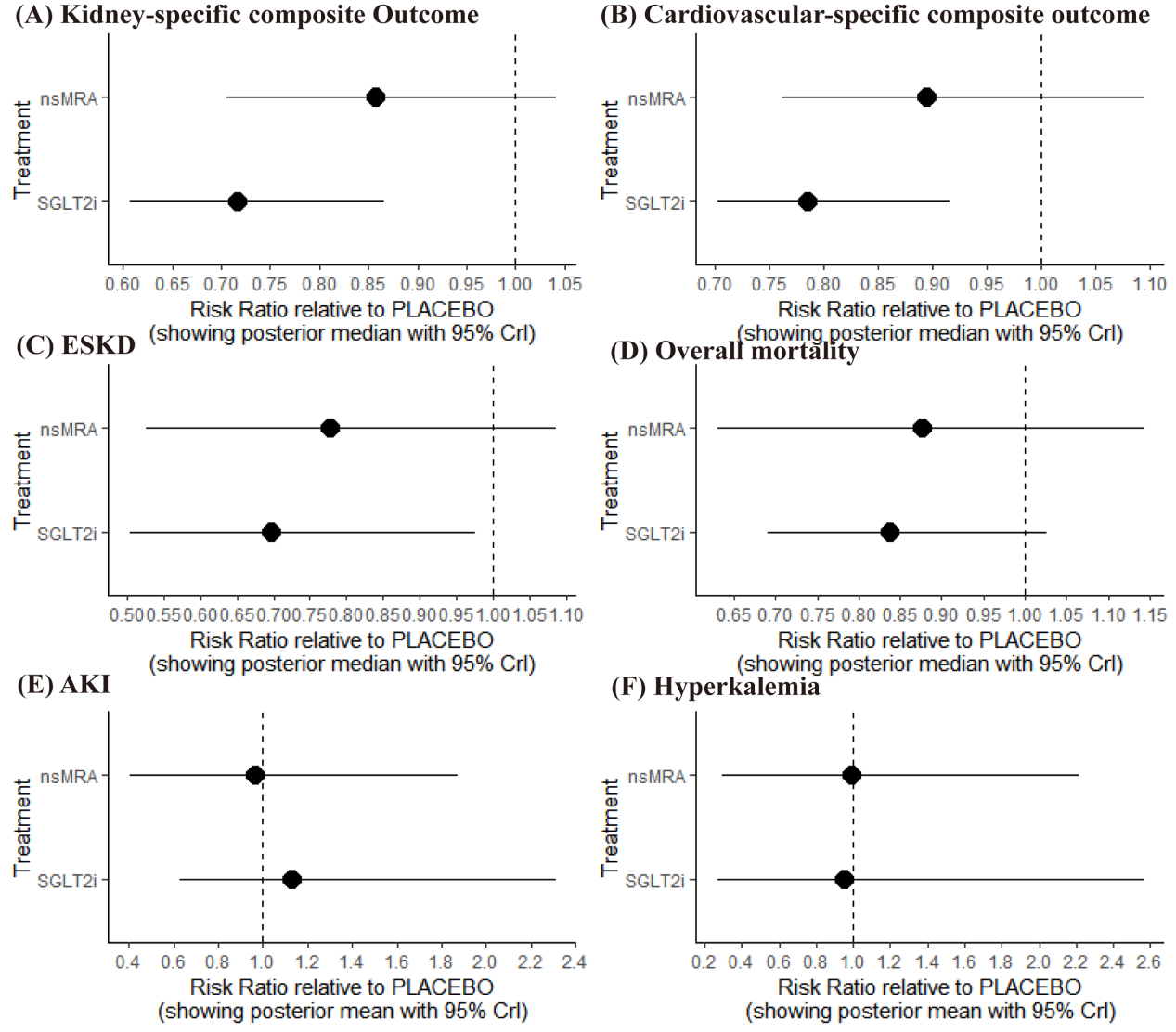


**Supplement Figure 2.|** A-F drawn forest plot for NMA. ESKD, End-stage kidney disease; AKI, Acute kidney injury；NMA, Network meta-analysis.
