# Supplementary material for: Comparative efficacy and safety of SGLT2is and ns-MRAs in patients with diabetic kidney disease: a systematic review and network meta-analysis
Source: Front Endocrinol (Lausanne). 2024 Jul 4;15:1429261. doi: 10.3389/fendo.2024.1429261 (PMC11256196; doi:10.3389/fendo.2024.1429261)
Supplement: Supplementary file 4 [file Table_1.docx]

| DAPA-CKD | ≥50% decrease in eGFR, ESKD (maintenance dialysis for ≥28 days, kidney transplantation, or an eGFR of <15 ml/min/1.73 m^2^ confirmed by a second measurement after ≥28 days), death from renal causes. |
| --- | --- |
| CREDENCE | ESKD (dialysis for ≥30 days, kidney transplantation, or an eGFR of <15 ml/min/1.73 m^2^ sustained for ≥30 days according to central laboratory assessment), doubling of the serum creatinine level from baseline sustained for ≥30 days according to central laboratory assessment, or death from renal disease. |
| CANVAS Program | ESKD, renal death, ≥40% reduction in eGFR or doubling of serum creatinine. |
| SCORED | ≥50% decrease in eGFR, long-term dialysis, renal transplantation, or an eGFR of <15 ml/min/1.73 m^2^ for ≥30 days. |
| ARTS-DN | ≥40% decrease in eGFR |
| FIDELIO-DKD | ≥40% decrease in eGFR |
| FIGARO-DKD | kidney failure (the initiation of chronic dialysis for ≥90 days or kidney transplantation), an eGFR of <15 ml/min/1.73 m^2^ for ≥30 days),≥ 40% reduction in eGFR for ≥28 days, or death from renal causes. |

**Table S1. Definition of kidney composite outcomes across included trials**

DAPA-CKD, Dapagliflozin and Prevention of Adverse Outcomes in Chronic Kidney Disease; CREDENCE, Canagliflozin and Renal Events in Diabetes with Established Nephropathy Clinical Evaluation; CANVAS, Canagliflozin Cardiovascular Assessment Study; EMPA‐REG OUTCOME, Empagliflozin, Cardiovascular Outcomes, and Mortality in Type 2 Diabetes; SCORED, Sotagliflozin Cardiovascular Outcomes Trial in Type 2 Diabetes Mellitus in Patients with Chronic Kidney Disease and Cardiovascular Disease; ARTS-DN, Advanced Renal Therapeutics Study of Diabetic Nephropathy; FIDELIO-DKD, Finerenone in Reducing Kidney Disease and Heart Failure Events in Patients with Type 2 Diabetes Mellitus and Chronic Kidney Disease; JapicCTI173695, Efficacy and Safety of Esaxerenone for the Treatment of Type 2 Diabetes with Microalbuminuria; ESAX-DN, Evaluation of the Effects of Esaxerenone on Renal and Cardiovascular Outcomes in Patients with Type 2 Diabetes Mellitus and Nephropathy; FIGARO-DKD, Finerenone in Reducing Kidney Failure and Disease Progression in Diabetic Kidney Disease; eGFR, Estimated glomerular filtration rate; ESKD, End-stage kidney disease.
