# Supplementary material for: Comparative efficacy and safety of SGLT2is and ns-MRAs in patients with diabetic kidney disease: a systematic review and network meta-analysis
Source: Front Endocrinol (Lausanne). 2024 Jul 4;15:1429261. doi: 10.3389/fendo.2024.1429261 (PMC11256196; doi:10.3389/fendo.2024.1429261)
Supplement: Supplementary file 5 [file Table_2.docx]

| DAPA-CKD | hospitalization for heart failure or death from cardiovascular cause. |
| --- | --- |
| CREDENCE | nonfatal myocardial infarction, or nonfatal stroke, and a composite of hospitalization for heart failure or cardiovascular death. |
| CANVAS Program | cardiovascular death, nonfatal myocardial infarction, nonfatal stroke, hospitalized heart failure. |
| EMPA-REG OUTCOME | the first occurrence of death from cardiovascular causes, nonfatal myocardial infarction, or nonfatal stroke. |
| SCORED | cardiovascular causes, hospitalizations for heart failure, and urgent visits for heart failure. |
| ARTS-DN | death from cardiovascular causes, nonfatal myocardial infarction, nonfatal stroke, or hospitalization for heart failure. |
| FIDELIO-DKD | death from cardiovascular causes, nonfatal myocardial infarction, nonfatal stroke, or hospitalization for heart failure. |
| FIGARO-DKD | death from cardiovascular causes, nonfatal myocardial infarction, nonfatal stroke, or hospitalization for heart failure. |

**Table S2. Definition of cardiovascular specific composite outcomes across included trials**
