# Supplementary material for: Comparative efficacy and safety of SGLT2is and ns-MRAs in patients with diabetic kidney disease: a systematic review and network meta-analysis
Source: Front Endocrinol (Lausanne). 2024 Jul 4;15:1429261. doi: 10.3389/fendo.2024.1429261 (PMC11256196; doi:10.3389/fendo.2024.1429261)
Supplement: Supplementary file 6 [file Table_3.docx]

**Table S3. Characteristics of Clinical Trials Included in the Network meta-analysis**

DAPA-CKD, Dapagliflozin and Prevention of Adverse Outcomes in Chronic Kidney Disease; CREDENCE, Canagliflozin and Renal Events in Diabetes with Established Nephropathy Clinical Evaluation; CANVAS, Canagliflozin Cardiovascular Assessment Study; EMPA‐REG OUTCOME, Empagliflozin, Cardiovascular Outcomes, and Mortality in Type 2 Diabetes; SCORED, Sotagliflozin Cardiovascular Outcomes Trial in Type 2 Diabetes Mellitus in Patients with Chronic Kidney Disease and Cardiovascular Disease; ARTS-DN, Advanced Renal Therapeutics Study of Diabetic Nephropathy; FIDELIO-DKD, Finerenone in Reducing Kidney Disease and Heart Failure Events in Patients with Type 2 Diabetes Mellitus and Chronic Kidney Disease; JapicCTI173695, Efficacy and Safety of Esaxerenone for the Treatment of Type 2 Diabetes with Microalbuminuria; ESAX-DN, Evaluation of the Effects of Esaxerenone on Renal and Cardiovascular Outcomes in Patients with Type 2 Diabetes Mellitus and Nephropathy; FIGARO-DKD, Finerenone in Reducing Kidney Failure and Disease Progression in Diabetic Kidney Disease; y, years. Sex indicates No. of male patients.

*Mean ± SD; Median (interquartile range).
